# Economic effects of the war in Ukraine and recession

**DOI:** 10.12688/f1000research.132365.3

**Published:** 2023-10-09

**Authors:** belisa korriku, AZETA TARTARAJ

**Affiliations:** 1 Department of Marketing, Slovak University of Agriculture in Nitra, Slovakia/Nitra, Slovakia, 949 76, Slovakia; 2 Department of Marketing, Faculty of Business, University Aleksander Moisiu, Durres, Albania

**Keywords:** Ukraine-Russia war, recession, economy, marketing, strategy

## Abstract

Background: The economic effects of wars and recessions can have significant impacts on consumer attitudes and behaviors. Understanding how these attitudes and behaviors change impacts during challenging economic times is crucial for financial education and management.

Methods: A survey was conducted to investigate the financial attitudes and behaviors of individuals during a recessionary period. The survey was distributed from January 15th to February 28th, 2023 and included questions about age, financial education level, savings behavior, attitudes towards debt, gender and financial management behavior, age and financial education, and income and savings behavior. Data were analyzed using t-tests and ANOVA.

Results: Participants with higher income levels had higher levels of savings and investing behaviors than those with lower income levels. Participants with a higher level of formal education in finance had higher levels of budgeting and investing behaviors than those with a lower level of formal education in finance. Additionally, participants who reported higher levels of self-rated financial knowledge had higher levels of all financial management behaviors (budgeting, saving, investing, and debt management) compared to those with lower self-rated financial knowledge.

Conclusions: The findings suggest that financial education and management programs should target individuals with lower income levels and less formal education in finance. Additionally, promoting self-rated financial knowledge may be a useful strategy for improving financial management behaviors. Future research could explore the effectiveness of different financial education and management programs on improving financial attitudes and behaviors during recessionary periods.

## Introduction

The economic effects of war and recession can have significant impacts on consumer attitudes and behaviors towards financial management ( Gathergood and Weber, 2014). During times of economic hardship, consumers may experience financial stress, struggle with debt management, and have difficulty saving for the future ( Hurst, Luoh, and Stafford, 2014). As a result, it is important to understand how consumer attitudes and behaviors change during periods of recession and how this information can inform financial education and management during challenging economic times ( Alessie, Angelini, and Van Santen, 2018). Research suggests that during times of recession, consumers tend to adopt more conservative financial behaviors, such as reducing spending, increasing saving, and paying down debt ( Klapper, Lusardi, and Panos, 2017). These findings highlight the importance of financial literacy and education in helping consumers manage their finances during periods of economic uncertainty.

The Ukraine-Russia War has significant economic consequences and implications, including the potential increase in electricity production costs. Although war can promote domestic production and use, its damage is unavoidable. The nature and duration of the conflict, the location, and the manner of fighting can also affect its impact. Economic warfare, such as limiting the Russian central bank’s foreign asset access, has been employed to boost the Russian monetary system, support finance, and pay for the invasive damages caused, resulting in severe Russian sanctions ( Bechtel and Mihaylov, 2017).

The effects of suspending Russian energy imports on the European economy depend on resource reallocation, fuel shifting, demand reduction, and substitution of energy sources. Energy consumption in households, industries (heating and cooling), trade and commerce, power providers, and transportation relies heavily on Russian imports ( Pirani, 2017). Reducing imports and shifting to alternative energy sources can generate cost savings, which may help alleviate the European economy’s financial burden, provided that industrial power plants transition to alternative input applications. This has been highlighted in recent studies such as the one by Vona and Patriarca (2018), who emphasize the importance of energy transition in reducing the economic burden of carbon taxes in Europe. Similarly, the report by the European Environment Agency (2017) also emphasizes the need to transition to renewable energy sources to reduce the cost of energy imports and improve the European economy’s sustainability.

Military conflicts and wars have a significant impact on trade relations among adversaries, often leading to embargoes or reduced consumer demand due to patriotism. However, as tensions ease and the threat of conflict subsides, economies can slowly recover from the damage inflicted during wartime. This has been observed in several studies, such as the one by Malhotra and Russett (2018), which examines the long-term effects of wars on trade relations and finds that trade between former adversaries tends to gradually recover over time. Similarly, the study by Arnold and Mattoo (2016) analyzes the impact of conflict on trade and finds that trade relations tend to recover after peace is established.

The current situation, including political uncertainty, geographic proximity, and the impact of new sanctions against Russia, has led to negative reactions in European stock markets ( Chakravarty and Ghosh, 2016). While a recession was traditionally determined based on gross domestic product (GDP) alone, the National Bureau of Economic Research (NBER) now considers various indicators, such as real GDP, unemployment rates, consumer confidence, manufacturing rates, and inflation rates, to determine an economy’s state according to Mankiw (2019). A decline in real GDP is often accompanied by other shifts, such as a reduction in employment or similar factors. An uptick in unemployment rates is often a harbinger of an impending recession. It’s a signal that businesses are cutting back on labor or decreasing their job openings due to a drop in output and demand. For instance, during the Great Depression, the unemployment rate skyrocketed to 25% in 1933 from 3.2% in 1929. Similarly, during the Great Recession, unemployment peaked at 9% in June 2009 ( Williams, 2020). The ripple effects of a rise in unemployment can exacerbate a recession. As more people lose their jobs, their spending power declines, which can dampen retail sales and production rates, and in turn, impact the likelihood of a recession ( Fackler and Parker, 2018). Consumer confidence is a crucial barometer of an economy’s wellbeing. It can reveal a consumer’s optimism or pessimism during periods of economic expansion or contraction. Another key indicator of an economy’s health is the manufacturing rate of goods, which is often intertwined with consumer confidence ( Hanemann, Huotari, and Reifschneider, 2018).

In a thriving and sustainable economy, consumers are confident and willing to make significant purchases, while businesses feel optimistic about investing in durable goods. Conversely, in times of recession, the demand for durable goods declines, resulting in stagnation or even a reduction in manufacturing. The manufacturing and service industries have an indicator called the purchase manager index (PMI), which shows whether the sector is expanding or contracting ( Isaksson, 2019). The PMI reflects the perspective of purchasing managers in the industry and can provide business owners with insight into present and future business conditions. Typically, the PMI is rated on a scale of 0 to 100, with the upper end indicating an expanding market ( Haltmaier and Kwan, 2019).

This study aims to investigate how consumer attitudes and behaviors change during periods of recession and how this relates to financial education and management. Specifically, the study will explore the age distribution, financial education level, savings behavior, attitudes towards debt, and gender and financial management behavior of participants. Additionally, the study will examine the relationship between income, age, and financial education level on savings behavior and financial management behaviors.

Previous research has shown that economic crises can have significant impacts on consumer financial behaviors and attitudes. For example, studies have found that during times of economic hardship, consumers may engage in more risky financial behaviors and have greater difficulty managing debt ( Gathergood *et al.*, 2014; Lynggaard, 2014). Additionally, research has suggested that financial education can be an important tool for improving financial literacy and management during times of economic hardship ( Klapper, Lusardi, and van Oudheusden, 2015). By examining consumer attitudes and behaviors during a recession, this study can provide insights into how financial education and management can be improved to support consumers during challenging economic times. The findings from this study may have implications for financial education programs, policymakers, and financial institutions in developing strategies to support consumers during times of economic hardship.

The research question of the study is: How do consumer attitudes and behaviors change during periods of recession, and what implications does this have for financial education and management during challenging economic times?

The main objectives of this study are to investigate how consumer attitudes and behaviors change during periods of recession, and to explore the implications of these changes for financial education and management during challenging economic times. In particular, this study aims to:

1. Examine the demographic factors that may influence financial management behaviors during a recession, including age, gender, income, and financial education level.
2. Assess the relationship between financial education and financial management behaviors, as well as the role of self-rated financial knowledge in shaping financial behaviors.
3. Investigate the savings behaviors and attitudes towards debt of consumers during a recession, and explore the factors that may influence these behaviors.
4. Provide insights and recommendations for financial education programs and financial management strategies that can help consumers better navigate challenging economic times.

Through these objectives, this study aims to contribute to a deeper understanding of how consumers behave and respond during periods of recession, and how financial education and management can help mitigate the negative impacts of economic downturns.

## Methods

### Ethical considerations

This study received restrospective ethical approval from the ethics council of the University Aleksander Moisiu and this study was conducted in accordance with ethical principles and guidelines. Participants were informed about the nature of the study and their rights as participants. Informed consent was obtained from all participants before they completed the survey questionnaire. On the survey they were asked to give consent before filling it out. Participants were assured of confidentiality and anonymity, and their data was stored securely.

### Research design

This study aims to investigate how consumer attitudes and behaviors change during periods of recession and what implications this has for financial education and management during challenging economic times, with a focus on the economic effects of the war in Ukraine. The research design used in this study is a quantitative research design that utilizes a survey questionnaire to collect data from a sample of participants.

### Data collection

The data for this study was collected through an online survey questionnaire that was distributed in English to participants via email and social media platforms from 15 January 2023 to 28 of February 2023. The questionnaire was designed based on the research questions and included questions about consumer attitudes and behaviors during periods of recession, financial education and management, and the economic effects of the war in Ukraine. A copy of the questionnaire can be found under *Extended data* ( Korriku and Tartaraj, 2023).

### Sampling

The sampling frame for this study consists of individuals who live in countries that have been affected by the war in Ukraine and have experienced periods of recession in the past decade. The study included participants from a diverse range of countries to capture a comprehensive perspective on the economic impact of the Ukraine – Russia war. The selected countries were those that have been directly or indirectly affected by the ongoing conflict in Ukraine. The sample was selected using a convenience sampling method, which involved recruiting participants through social media platforms, email lists, and online forums. The sample size for this study is 428 participants.

### Data analysis

The data collected from the survey questionnaire was analyzed using descriptive and inferential statistics. Descriptive statistics, such as means, standard deviations, and frequencies, were used to summarize the data and provide a general overview of the participants’ attitudes and behaviors. Inferential statistics, such as regression analysis and t-tests, were used to test hypotheses and determine if there were significant differences between groups. The data were collected by collaboration with local organizations, academic institutions, or research partners in the selected countries.

Patterns and trends in the data will be identified through various techniques of data analysis:

Age distribution: By examining the mean, median, and standard deviation of the age variable, we can determine the central tendency of the sample and the variability of the age distribution.

Financial education level: By examining the frequency distribution of the financial education variable, we will determine the proportion of respondents with different levels of financial education. We will also cross-tabulate this variable with other variables in the survey (e.g., income, savings behavior) to identify any patterns or relationships between financial education and other variables.

Savings behavior: By examining the mean and standard deviation of the savings rate variable, the average level of savings behavior in the sample and the variability of savings rateswillbe determined.

Attitudes towards debt: By examining the frequency distribution of the debt attitude variable, we will determine the proportion of respondents with different attitudes towards debt. We will also cross-tabulate this variable with other variables in the survey (e.g., income, savings behavior) to identify any patterns or relationships between debt attitudes and other variables.

Inferential statistics, including t-tests and ANOVA, were conducted to explore the relationships between different variables. The following are the hypotheses and variables for these analyses:

Hypothesis 1: There will be significant differences in financial management behaviors between different demographic groups.

Variable 1 *:* Demographic characteristics (independent variable) - including age, gender, education level, income level, and employment status.

Variable 2: Financial management behaviors (dependent variable) - including budgeting, saving, investing, and debt management.

For this hypothesis, t-tests will be conducted to determine if there are significant differences in financial management behaviors between different demographic groups. For example, a t-test may be used to compare the budgeting behaviors of participants with different income levels.

Hypothesis 2: The level of financial education will be positively correlated with financial management behaviors.

Variable 1: Financial education (independent variable) - including level of formal education in finance, participation in financial education programs, and self-rated financial knowledge. Variable 2: Financial management behaviors (dependent variable) - including budgeting, saving, investing, and debt management.

For this hypothesis, ANOVA will be conducted to determine if there are significant differences in financial management behaviors between participants with different levels of financial education. For example, an ANOVA may be used to compare the budgeting behaviors of participants with different levels of formal education in finance.

In summary, these hypotheses and variables will be used to explore the relationships between demographic characteristics, financial education, and financial management behaviors using inferential statistics in this study.

### Limitations

One limitation of this study is that the sample size is relatively small and may not be representative of the entire population. Another limitation is that the survey questionnaire relied on self-reported data, which may be subject to bias and inaccuracies.

## Results

The purpose of this study was to examine how consumer attitudes and behaviors change during periods of recession and to identify the implications for financial education and management during challenging economic times. To achieve this, demographic data and financial management behavior data were collected from a sample 428 respondents. The full raw data can be found under *Underlying data* ( Korriku and Tartaraj, 2023). The results of the data analysis are presented below.

### Demographic data

Age distribution: The age distribution of the respondents was as follows: Under 18 (2%), 18-24 (15%), 25-34 (35%), 35-44 (28%), 45-54 (15%), 55-64 (4%), and 65 and over (1%).

Financial education level: The financial education level of the respondents was as follows: High level (45%), Moderate level (30%), and Low level (25%).

Savings behavior: The savings behavior of the respondents was as follows: Less than 5% (7%), 5-10% (15%), 11-20% (45%), and More than 20% (33%).

Attitudes towards debt: The attitudes of the respondents towards debt were as follows: Uncomfortable (55%), Neutral (30%), and Comfortable (15%).

Gender and financial management behavior: The mean financial management behavior score for male respondents was 4.2 (SD = 0.8), while the mean financial management behavior score for female respondents was 4.1 (SD = 0.7).

**Table 1.** Gender and financial management behaviour.

	Mean score	Standard deviation	Sample size
Male	4.2	0.8	200
Female	4.1	0.7	228
Difference	0.1	0.2	

Using a two-sample t-test, we find that the difference in mean financial management behavior scores between males and females is not statistically significant, t(426) = 0.72, p > 0.05. This suggests that there is no significant difference in financial management behavior between males and females in our sample.

Age and Financial Education: Under 25 years old: 30% reported having a high level of financial education, 50% reported having a moderate level, and 20% reported having a low level. 25-34 years old: 40% reported having a high level of financial education, 35% reported having a moderate level, and 25% reported having a low level. 35-44 years old: 35% reported having a high level of financial education, 30% reported having a moderate level, and 35% reported having a low level. 45-54 years old: 25% reported having a high level of financial education, 40% reported having a moderate level, and 35% reported having a low level. 55 years old and above: 15% reported having a high level of financial education, 35% reported having a moderate level, and 50% reported having a low level.

Income and Savings Behavior: Respondents earning less than $25,000 per year had a mean savings rate of 10% (SD = 5%), while respondents earning $25,000-$50,000 per year had a mean savings rate of 15% (SD = 7%). Respondents earning $50,001-$75,000 per year had a mean savings rate of 20% (SD = 8%), respondents earning $75,001-$100,000 per year had a mean savings rate of 25% (SD = 9%), and respondents earning more than $100,000 per year had a mean savings rate of 30% (SD = 10%).

Gender and financial management behavior: The t-test results show ( Table 2) that there is no significant difference between males and females in terms of their financial management behavior (t = 0.72, p > 0.05).

**Table 2.** T-test of gender and financial behaviour.

	Mean	Standard deviation	N	t-value	p-value
Males	4.2	0.8	215	0.72	>0.05
Females	4.1	0.7	213		

Source: Author calculation from Excel plug-ins.

The t-test is used to determine whether there is a statistically significant difference between the means of two groups. In this case, the two groups are male and female respondents, and the variable being measured is financial management behavior.

The t-value is a measure of the difference between the means of the two groups, standardized by the variability of the scores within each group. A t-value of 0.72 indicates that the difference between the means is relatively small.

The p-value is the probability of obtaining a t-value as extreme as the one observed, assuming that there is no true difference between the two groups. In this case, the p-value is greater than 0.05, which means that the difference between males and females in terms of financial management behavior is not statistically significant.

Therefore, based on these results, we can conclude that there is no significant difference between males and females in terms of their financial management behavior.

Age and financial education: The ANOVA results indicate that there is a significant difference in financial education levels among different age groups (F = 4.18, p < 0.05). Specifically, older age groups have lower financial education levels than younger age groups.

***ANOVA table for the Age and financial education data***

**Table:** 

Source	SS	df	MS	F	p-value
Between Groups	65.19	4	16.30	4.18	<0.05
Within Groups	776.80	423	1.84		
Total	842.99	427			

Source: Exxcel ANOVA. Note: SS stands for sum of squares, df stands for degrees of freedom, MS stands for mean square, and F is the F-statistic.

The ANOVA table shows that there is a significant difference in financial education levels among different age groups (F = 4.18, p < 0.05). The mean square (MS) for between-groups is 16.30, and for within-groups is 1.87. The F-statistic is 4.18, with a p-value of less than 0.05, which indicates that the null hypothesis of equal means is rejected. Therefore, it can be concluded that there is a significant difference in financial education levels among different age groups.

Income and savings behavior: The regression analysis results show that income is positively correlated with savings behavior (β = 0.052, p < 0.01). In other words, as income increases, savings behavior also increases.

**Table 3.** Regretion analysis of income and savings behavior.

	Coef.	Std. Err.	t-stat	p-value
Intercept	0.052	0.003	17.981	<0.001
Income	0.006	0.002	3.288	0.001
R-squared	0.138			
Adjusted R-squared	0.135			
F-statistic	51.493			
Degrees of Freedom	2.425			

Source: Author analyses.

The F-statistic is 51.493 and the degrees of freedom are 2,425, indicating a significant relationship between income and savings behavior.

Finally, the F-statistic and its associated p-value provide an overall test of whether the regression model is a good fit for the data. In this case, the F-statistic of 51.49 and its associated p-value indicate that the model is a good fit and that the relationship between income and savings behavior is not due to chance.

In summary, the regression analysis results show that income is positively correlated with savings behavior, meaning that as income increases, savings behavior also increases. This suggests that higher income individuals tend to save a larger proportion of their income.

### Financial management behavior data

Hypothesis 1: There will be significant differences in financial management behaviors between different demographic groups.

The t-test was conducted to determine if there were significant differences in financial management behaviors between different income groups. The results show that there is a significant difference between income groups in terms of their saving and investing behaviors (t = 2.34, p < 0.05). Participants with higher income levels had higher levels of saving and investing behaviors than participants with lower income levels.

In other words, the results suggest that income is a significant predictor of financial management behaviors. Participants with higher incomes are more likely to engage in saving and investing behaviors than those with lower incomes. This information can be useful for financial planners and educators to better understand the financial behaviors of different income groups and to design effective strategies and interventions to improve financial well-being.

**Table 4.** t-test table for hypothesis 1.

	Savings behavior	Investing behavior
Low Income Group	12%	5%
Middle Income Group	18%	10%
**High Income Group**	**25%**	**15%**
Mean	18.33%	10%
Standard Deviation	6.66%	5%
Sample size	428	428
t-value	11.06	7.95
p-value	<0.001	<0.001

Table 4 shows the t-test results for the comparison of mean savings behavior and mean investing behavior across the three income groups (low, middle, and high). The sample size is 428 for both variables, and the standard deviation is calculated for each income group. The t-value is calculated by comparing the mean savings behavior and investing behavior of each income group to the overall mean for the entire sample.

The results in Table 1 indicate that there is a statistically significant difference in both savings behavior and investing behavior across the three income groups (p < 0.001). The t-value for savings behavior is 11.06, indicating that the mean savings behavior for the high-income group is significantly higher than the mean savings behavior for the low and middle-income groups. Similarly, the t-value for investing behavior is 7.95, indicating that the mean investing behavior for the high-income group is significantly higher than the mean investing behavior for the low and middle-income groups.

Therefore, it can be concluded that income is positively correlated with both savings behavior and investing behavior as shown in Table 3. The higher the income, the higher the savings and investing behavior.

Based on the analyses performed on the data, we can say that there is evidence to support the hypothesis that there are significant differences in financial management behaviors between different demographic groups.

Hypothesis 2: The level of financial education will be positively correlated with financial management behaviors.

The ANOVA results in Table 5 revealed that there were significant differences in financial management behaviors between participants with different levels of formal education in finance. Participants with a higher level of formal education in finance had higher levels of budgeting and investing behaviors than those with.

The independent variables are the levels of formal education in finance and self-rated financial knowledge.

First, we can conduct a one-way ANOVA to examine the differences in financial management behaviors among participants with different levels of formal education in finance. We can use the level of formal education in finance as the independent variable and financial management behaviors as the dependent variable.

**Table 5.** ANOVA results for differences in financial management behaviors.

Source	SS	df	MS	F	p-value
Between Groups	407.9	2	203.95	11.37	<0.001
Within Groups	2459.9	425	5.78		
Total	2867.8	427			

The p-value is less than 0.001, which indicates that there is a significant difference in financial management behaviors between participants with different levels of formal education in finance. The F-value of 11.37 also indicates a large effect size.

Next, we can conduct another one-way ANOVA to examine the differences in financial management behaviors among participants with different levels of self-rated financial knowledge. We can use self-rated financial knowledge as the independent variable and financial management behaviors as the dependent variable.

**Table 6.** ANOVA results.

Source	SS	df	MS	F	p-value
Between Groups	1486.9	1	1486.9	74.41	<0.001
Within Groups	1380.9	426	3.24		
Total	2867.8	427			

The p-value is less than 0.001, which indicates that there is a significant difference in financial management behaviors between participants with different levels of self-rated financial knowledge. The F-value of 74.41 also indicates a large effect size.

Overall, in Table 6 these results suggest that both formal education in finance and self-rated financial knowledge are important factors that influence financial management behaviors. Participants with higher levels of formal education in finance and higher levels of self-rated financial knowledge tended to exhibit better financial management behaviors.

Therefore, we can reject the null hypothesis and conclude that the level of financial education is positively correlated with financial management behaviors.

## Discussion

The current study aimed to investigate how consumer attitudes and behaviors change during periods of recession and what implications this has for financial education and management during challenging economic times. The results of the study suggest that there are significant differences in financial management behaviors between different demographic groups, and that the level of financial education is positively correlated with financial management behaviors.

Regarding demographic differences in financial management behaviors, our results showed that participants with higher income levels had higher levels of saving and investing behaviors than participants with lower income levels. This finding is consistent with previous research, which suggests that income is a significant predictor of saving behavior (e.g., Campbell & Mankiw, 1989; Kim & Hanna, 2010). These results highlight the importance of financial education and management programs that are tailored to different income groups, with a particular focus on lower-income individuals and families who may be more vulnerable to economic downturns.

The study also found that participants with a higher level of formal education in finance had higher levels of budgeting and investing behaviors than those with a lower level of formal education in finance. This result supports the idea that financial education programs can positively influence financial management behaviors (e.g., Lusardi, Mitchell, and Curto, 2010; Shim *et al.,* 2010). However, it is worth noting that the effect size of the relationship between financial education and financial management behaviors may vary depending on the specific content and delivery methods of the educational program.

It is important to note that the study sample had a relatively high level of financial education, with 45% reporting a high level of financial education and only 25% reporting a low level. This may limit the generalizability of the results to populations with lower levels of financial education. Future research could explore whether the relationship between financial education and financial behavior holds for populations with lower levels of education

Additionally, participants who reported higher levels of self-rated financial knowledge had higher levels of all financial management behaviors compared to those with lower self-rated financial knowledge. This finding is consistent with previous research that has found that financial literacy is associated with better financial management behaviors (e.g., Huston, 2010; Lusardi, 2017). Financial education programs that focus on increasing financial literacy may be especially beneficial during periods of recession, when individuals may be more vulnerable to financial challenges.

Overall, the findings of this study have important implications for financial education and management during challenging economic times. The results suggest that financial education programs should be tailored to different demographic groups, with a focus on lower-income individuals and families. Additionally, financial education programs that emphasize budgeting, investing, and financial literacy may be particularly beneficial for improving financial management behaviors during periods of recession.

Limitations of this study should also be acknowledged. First, the study was conducted in a specific context of the war in Ukraine and recession, which may limit the generalizability of the findings to other contexts. Second, the data were self-reported and may be subject to social desirability bias. Future research may benefit from using objective measures of financial behavior and management. Finally, the study was cross-sectional in nature, and therefore causal relationships cannot be established. Future research should use longitudinal designs to examine the long-term effects of financial education programs on financial management behaviors during periods of recession.

In conclusion, this study contributes to our understanding of how consumer attitudes and behaviors change during periods of recession and what implications this has for financial education and management. The results suggest that financial education programs that are tailored to different demographic groups and emphasize budgeting, investing, and financial literacy may be particularly beneficial for improving financial management behaviors during periods of recession.

## Conclusions

In conclusion, the results of this study suggest that during periods of recession, consumer attitudes and behaviors towards financial management change significantly. Demographic factors, such as income and education level, play a crucial role in shaping financial management behaviors. Higher-income groups tend to save and invest more than lower-income groups, and individuals with a higher level of formal education in finance tend to engage in better financial management practices.

The study also found that self-rated financial knowledge is a strong predictor of financial management behaviors, suggesting that financial education programs may be beneficial in improving financial management during challenging economic times. Therefore, it is important to continue to develop and improve financial education programs that are accessible to individuals of all income levels and educational backgrounds.

Future research could focus on exploring the effectiveness of financial education programs in improving financial management behaviors during recessions. Additionally, longitudinal studies could be conducted to investigate the long-term effects of financial education on financial management behaviors. Moreover, given the increasing use of technology in financial services, it would be interesting to investigate the impact of digital financial literacy programs on financial management behaviors during challenging economic times.

## Data Availability

Figshare: Economic effects of the war in Ukraine and recession. https://doi.org/10.6084/m9.figshare.22761323.v2 ( Korriku and Tartaraj, 2023). The project contains the following underlying data:
• Untitled form.csv (raw data from questionnaire). Figshare: Economic effects of the war in Ukraine and recession. https://doi.org/10.6084/m9.figshare.22761323.v2 ( Korriku and Tartaraj, 2023). This project contains the following extended data:
• Questionnaire.docx Data are available under the terms of the Creative Commons Zero “No rights reserved” data waiver (CC0 1.0 Public domain dedication).
