# Supplementary material for: Economic effects of the war in Ukraine and recession
Source: F1000Res. 2023 Oct 9;12:525. Originally published 2023 May 22. [Version 3] doi: 10.12688/f1000research.132365.3 (PMC10811416; doi:10.12688/f1000research.132365.3)
Supplement: Peer review history

# Reviewer report: Reviewer response for version 3

**DOI:** 10.5256/f1000research.157031.r236873

**Published:** 2024-01-30

**Authors:** Ruth Endam Mbah

**Affiliations:** 1 Department of Business Administration, McPherson College, McPherson, Kansas, USA

This research has great potential if structured better. The authors have done a great job so far but need to revise the work to make it better. Below are my comments and suggestions:

 1) The title of this paper does not seem to correlate with its content. Reading through the paper I see no connection between the purpose of the study and the Ukraine war. From my reading, I think the paper seems to address consumer behavior during adverse economic times like recession and the impact of such behaviors on financial management.  I will suggest rewording the title and abstract to reflect the actual purpose of the paper.

 2) The paper starts by introducing the impact of the Ukraine-Russia war and then moves to consumer behavior in times of recession and financial management/education. There is a significant disconnect. As a reader, I feel lost at this point as to what exactly is the focus of the paper and what connection the war has to the entire manuscript. 

 3) I am not a statistician and would leave the method part to a seasoned statistician. However, I would like to see which countries were included in the study. The entire world has been affected by this war one way or the other and most countries have gone through recession at a given point. Hence, it will be great to know the exact countries involved in the study. As the authors said in their limitation section, I believe the sample size is small if you are talking about several countries. I would have loved to see justification as to why they chose the diverse tests to analyze each variable by backing up their choices and also referencing other authors who used such methods. This makes your methodology more convincing and addresses the replicability/reliability issue. I will leave this to a statistician to address. 

 I believe this study has potential if revised and restructured.

Is the work clearly and accurately presented and does it cite the current literature?

Partly

If applicable, is the statistical analysis and its interpretation appropriate?

I cannot comment. A qualified statistician is required.

Are all the source data underlying the results available to ensure full reproducibility?

Partly

Is the study design appropriate and is the work technically sound?

Partly

Are the conclusions drawn adequately supported by the results?

Yes

Are sufficient details of methods and analysis provided to allow replication by others?

Partly

Reviewer Expertise:

Debt Management, Student Debt, Higher Education, Public Policy, Financial Management, International Conflicts, International Relations

I confirm that I have read this submission and believe that I have an appropriate level of expertise to confirm that it is of an acceptable scientific standard, however I have significant reservations, as outlined above.

# Reviewer report: Reviewer response for version 3

**DOI:** 10.5256/f1000research.157031.r236869

**Published:** 2024-01-25

**Authors:** Imelda Sejdini

**Affiliations:** 1 Alekander Xhuvani University Elbasan, Elbasan, Albania

- The paper titled "Economic Effects of the War in Ukraine and Recession" presents a comprehensive analysis of the impact of economic turmoil and the Ukraine-Russia war on consumer financial behaviors and attitudes. The study’s primary focus is to understand how consumer attitudes and behaviors change during recessions and to assess the role of financial education in managing these challenges.
- The study highlights that higher-income individuals tend to have better saving and investing behaviors. Similarly, a higher level of formal education in finance correlates with improved budgeting and investing behaviors.
- A crucial finding is the positive correlation between self-rated financial knowledge and financial management behaviors. This underscores the importance of financial literacy and education in helping consumers navigate economic uncertainties.
- The paper delves into how the Ukraine-Russia war and subsequent economic repercussions have influenced consumer financial behaviors in Europe, emphasizing the need for adaptability in financial management practices during such times.
- The research employs a quantitative approach, analyzing data from a survey of 428 respondents across various countries affected by the war. This method provides a broad perspective on the economic impact of the conflict.
- The study suggests the need for tailored financial education programs targeting different demographic groups, especially focusing on lower-income individuals and those with less formal education in finance.
- The findings could inform policymakers and financial institutions in developing strategies to support consumers during economic hardships, emphasizing the role of financial literacy.
- The paper recommends exploring the effectiveness of different financial education programs during recessions and the impact of digital financial literacy on financial management.
- The relatively small and specific sample might limit the generalisability of the findings.
- The study's cross-sectional design precludes establishing causal relationships, suggesting the need for longitudinal studies in the future.
- Overall, this paper provides valuable insights into the dynamics of financial management behaviors during economic downturns, with a particular focus on the effects of the Ukraine-Russia war. It highlights the critical role of financial education and literacy in navigating economic challenges and suggests practical applications for policymakers and educators. While acknowledging its limitations, the study opens avenues for further research and underscores the importance of adaptable financial strategies in the face of global crises.

Is the work clearly and accurately presented and does it cite the current literature?

Yes

If applicable, is the statistical analysis and its interpretation appropriate?

Yes

Are all the source data underlying the results available to ensure full reproducibility?

Yes

Is the study design appropriate and is the work technically sound?

Yes

Are the conclusions drawn adequately supported by the results?

Yes

Are sufficient details of methods and analysis provided to allow replication by others?

Yes

Reviewer Expertise:

Business management, Business ethics, Reward management, Risk management and insurance, Human resources management.

I confirm that I have read this submission and believe that I have an appropriate level of expertise to confirm that it is of an acceptable scientific standard.

# Reviewer report: Reviewer response for version 3

**DOI:** 10.5256/f1000research.157031.r213058

**Published:** 2023-11-28

**Authors:** Michele Pigliucci

**Affiliations:** 1 Link Campus University, Rome, Italy

The article is interesting, but the author don't provide enough data about the involved respondents (countries where they are referred to, in particular). For this reason, I find the article incomplete

Is the work clearly and accurately presented and does it cite the current literature?

Yes

If applicable, is the statistical analysis and its interpretation appropriate?

Partly

Are all the source data underlying the results available to ensure full reproducibility?

Yes

Is the study design appropriate and is the work technically sound?

Partly

Are the conclusions drawn adequately supported by the results?

Partly

Are sufficient details of methods and analysis provided to allow replication by others?

Partly

Reviewer Expertise:

NA

I confirm that I have read this submission and believe that I have an appropriate level of expertise to confirm that it is of an acceptable scientific standard, however I have significant reservations, as outlined above.

# Response

**Published:** 2023-11-28

**Authors:** belisa korriku

**Affiliations:** Department of Marketing, Slovak University of Agriculture in Nitra, Nitra/Slovakia, slovakia, Slovakia

The study included participants from a diverse range of countries, including Ukraine, Russia, Belarus, Poland, Hungary, Romania, Moldova, and several Baltic states, to capture a comprehensive perspective on the economic impact of the Ukraine-Russia war. These selected countries were those that have been directly or indirectly affected by the ongoing conflict in Ukraine. Within these countries, specific regions and areas were identified for data collection. These regions were chosen based on their proximity to conflict zones or their known vulnerability to economic challenges resulting from the war. The data were collected through collaboration with local organizations, academic institutions, or research partners in the selected countries

# Reviewer report: Reviewer response for version 3

**DOI:** 10.5256/f1000research.157031.r213057

**Published:** 2023-10-10

**Authors:** Max Gillman

**Affiliations:** 1 Department of Economics, University of Missouri-St Louis, St. Louis, Missouri, USA

Seems to have responded to Reviewers.

Is the work clearly and accurately presented and does it cite the current literature?

Yes

If applicable, is the statistical analysis and its interpretation appropriate?

I cannot comment. A qualified statistician is required.

Are all the source data underlying the results available to ensure full reproducibility?

Yes

Is the study design appropriate and is the work technically sound?

Yes

Are the conclusions drawn adequately supported by the results?

Yes

Are sufficient details of methods and analysis provided to allow replication by others?

Yes

Reviewer Expertise:

Macroeconomics, monetary economics, business cycles, asset pricing, human capital.

I confirm that I have read this submission and believe that I have an appropriate level of expertise to confirm that it is of an acceptable scientific standard.

# Reviewer report: Reviewer response for version 2

**DOI:** 10.5256/f1000research.153868.r203343

**Published:** 2023-09-29

**Authors:** Michele Pigliucci

**Affiliations:** 1 Link Campus University, Rome, Italy

The geographical distribution of data is needed or, at least, the indication of what author means when says that respondents come from "countries that have been affected by the war".

Is the work clearly and accurately presented and does it cite the current literature?

Yes

If applicable, is the statistical analysis and its interpretation appropriate?

Partly

Are all the source data underlying the results available to ensure full reproducibility?

Yes

Is the study design appropriate and is the work technically sound?

Partly

Are the conclusions drawn adequately supported by the results?

Partly

Are sufficient details of methods and analysis provided to allow replication by others?

Partly

Reviewer Expertise:

Economic Geography, Geopolitics, regional development

I confirm that I have read this submission and believe that I have an appropriate level of expertise to confirm that it is of an acceptable scientific standard, however I have significant reservations, as outlined above.

# Reviewer report: Reviewer response for version 2

**DOI:** 10.5256/f1000research.153868.r203342

**Published:** 2023-09-04

**Authors:** Max Gillman

**Affiliations:** 1 Department of Economics, University of Missouri-St Louis, St. Louis, Missouri, USA

Yes it is better, thank you.

Is the work clearly and accurately presented and does it cite the current literature?

Yes

If applicable, is the statistical analysis and its interpretation appropriate?

I cannot comment. A qualified statistician is required.

Are all the source data underlying the results available to ensure full reproducibility?

Yes

Is the study design appropriate and is the work technically sound?

Yes

Are the conclusions drawn adequately supported by the results?

Yes

Are sufficient details of methods and analysis provided to allow replication by others?

Yes

Reviewer Expertise:

Macroeconomics, monetary economics, business cycles, asset pricing, human capital.

I confirm that I have read this submission and believe that I have an appropriate level of expertise to confirm that it is of an acceptable scientific standard.

# Reviewer report: Reviewer response for version 1

**DOI:** 10.5256/f1000research.145279.r175294

**Published:** 2023-07-05

**Authors:** Max Gillman

**Affiliations:** 1 Department of Economics, University of Missouri-St Louis, St. Louis, Missouri, USA

The topic is interesting. The problem is that the authors purport to show what happens during recessions to consumer savings and financial decisions, but then use a panel data study of 428 participants who have been affected by the war in Ukraine and have experienced recessions in the past decade. The sample is taken during the Ukraine wartime period of early 2023.

 Lumping together those affected by the Ukraine-Russia war, and those who have experienced a recession in the last decade is an odd combination. It is unclear what is the relation between those affected by the war and those experiencing a past recession. Therefore the results cannot be clearly delineated as to whether this is a wartime result or a recession result, which the authors claim.

 The results make perfect sense in general, that higher income people have a more sophisticated financial education. But I do not see how this relates to people affected by the war.

 This sort of question about behavior during recessions should not have anything to do with wartime periods unless there is some different type of hypothesis. Wartime of course creates economic calamity, more of what economists call a negative rare event. This is not a recession, but an economic disaster of untold proportions.

 The paper needs to somehow untangle the two vastly different effects: being affected by a current ongoing war, and how people behave in recessions in terms of savings and financial decisions.

 It is also unclear how the sample selection may bias the results: who would be likely to respond to such a study questionnaire? It may be those with a low value of time, or those particularly affected by the war. Either can bias the results, in various directions.

Is the work clearly and accurately presented and does it cite the current literature?

Yes

If applicable, is the statistical analysis and its interpretation appropriate?

I cannot comment. A qualified statistician is required.

Are all the source data underlying the results available to ensure full reproducibility?

Yes

Is the study design appropriate and is the work technically sound?

Yes

Are the conclusions drawn adequately supported by the results?

Yes

Are sufficient details of methods and analysis provided to allow replication by others?

Yes

Reviewer Expertise:

Macroeconomics, monetary economics, business cycles, asset pricing, human capital.

I confirm that I have read this submission and believe that I have an appropriate level of expertise to confirm that it is of an acceptable scientific standard, however I have significant reservations, as outlined above.

# Reviewer report: Reviewer response for version 1

**DOI:** 10.5256/f1000research.145279.r176655

**Published:** 2023-07-04

**Authors:** Michele Pigliucci

**Affiliations:** 1 Link Campus University, Rome, Italy

The article provide interesting results of a survey aiming at investigating how personal financial attitudes and behaviors change during crisis periods, namely Russo-Ukrainian war.

 The results of the survey are organised and summarised in order to give evidence to the different behaviors registered among people according to demographic and financial education elements.

 The article is well documented, and result well explained. Furthermore, an explanation about the geographic distribution of sampling could be needed to better read the result meaning.

 The indication about "countries that have been affected by the war in Ukraine and have experienced periods of recession in the past decade" looks too much general: every country is actually affected by the war, in different ways.

 Different countries register different levels of damage from Ukraine-Russo war, and it can change the result interpretation of the survey.

Is the work clearly and accurately presented and does it cite the current literature?

Yes

If applicable, is the statistical analysis and its interpretation appropriate?

Partly

Are all the source data underlying the results available to ensure full reproducibility?

Yes

Is the study design appropriate and is the work technically sound?

Partly

Are the conclusions drawn adequately supported by the results?

Partly

Are sufficient details of methods and analysis provided to allow replication by others?

Partly

Reviewer Expertise:

Economic Geography, Geopolitics, regional development

I confirm that I have read this submission and believe that I have an appropriate level of expertise to confirm that it is of an acceptable scientific standard, however I have significant reservations, as outlined above.
